# Predicting novice dental students' performances in conventional simulation: A prospective pilot study using haptic exercises

**DOI:** 10.1016/j.jds.2024.10.023

**Published:** 2024-11-12

**Authors:** Octave Nadile Bandiaky, Valériane Loison, Serena Lopez, Fabrice Pirolli, Christelle Volteau, Ludovic Hamon, Assem Soueidan, Laurent Le Guehennec

**Affiliations:** a Nantes University, Oniris, Univ Angers, CHU Nantes, INSERM, Regenerative Medicine and Skeleton, Nantes, France; b Le Mans University, Nantes Educational Research Center, CREN, Nantes, France; c Nantes University Hospital Center, Competence Center for Rare Oral and Dental Diseases, Nantes, France; d Nantes University, CHU Nantes, Research and Innovation Directiont, Methodology and Biostatistics Platform, Nantes, France; e Le Mans University, Computer Science Laboratory of Le Mans University, Le Mans, France; f Department of Periodontology, Faculty of Dental Surgery, University of Nantes, Nantes, France; g Department of Prosthodontic, Faculty of Dental Surgery, University of Nantes, Nantes, France

**Keywords:** Dental education, Haptic simulator, Manual dexterity, Student performance prediction, Student perception, Virtual reality

## Abstract

**Background/purpose:**

While haptic simulators in preclinical dentistry show promise, few studies predict novice dental students' performance in conventional simulations using haptic exercises. This study aimed to explore associations between (i) the number of failures in haptic exercises, (ii) the haptic performance index, and (iii) the quality of prosthetic preparation for cast crowns. Additionally, the students' perceptions regarding the use of the VirTeaSy Dental® haptic simulator was analyzed.

**Materials and methods:**

Forty novice students were randomly selected from the Dental Faculty of Nantes University in September 2022 (mean age: 19.7 ± 1.8 years). They completed four haptic exercises using the VirTeaSy Dental® simulator and prepared cast crowns on pedagogical phantom-mounted models. Data on haptic variables, prosthetic preparation quality scores, and the number of failed/successful haptic exercises were collected. Correlation analyses were conducted, and the mean preparation quality score was compared between students who failed and those who passed the haptic exercises. A questionnaire assessing the students' perceptions when using VirTeaSy Dental® was completed.

**Results:**

A correlation was found between the number of haptic exercise failures and the prosthetic preparation quality score, with students who failed showing lower scores (10.66 ± 3.69) compared to those who passed (13.72 ± 4.76) (*P* < 0.05). No correlation was observed for the haptic performance index. Students reported that the VirTeaSy simulator positively impacted their learning of milling gestures.

**Conclusion:**

The number of haptic exercise failures can predict performance in conventional simulations and help identify students with manual dexterity issues, guiding personalized preclinical training adjustments.

## Introduction

Conventional simulation using phantom heads constitutes a pivotal stage in the training program for dental students. Given the irreversible nature of most dental procedures, it is imperative for students to get the required skills for ensuring both safety and the procedural excellence.^1^ Therefore, during pre-clinical training, students extensively engage with simulators to hone their abilities, particularly for intricate procedures demanding precision and technical prowess.^1^^,^^2^ The genesis of conventional phantom head simulators traces back to Oswald Fergus in 1894, driven by the ambition in enhancing realism and closely replicate clinical scenarios.^2^^,^^3^ Over time, these simulators have undergone substantial advancements, equipping novice students with various motor skills. They facilitate the refinement of manual dexterity defined as the student's ability to skillfully use both hands in a coordinated manner to grasp and manipulate objects and make small and precise movements. With practice on phantom-mounted models, students improve their manual dexterity, becoming more and more precise in their gestures. This precision characterizes their pre-clinical performance, which is reflected in the quality of their dental preparations. These conventional simulators enable also the internalization of operational protocols in view of clinical practice.^4^, ^5^, ^6^

Like many fields within healthcare, dental simulation is continuously evolving, propelled by the advancements in digital technology. Particularly, the progress made in virtual reality and haptic technologies have fostered the use of innovative pedagogical strategies integrating haptic simulators into dental education.^7^ This convergence has ushered in a novel approach to acquiring technical skills in dentistry. A recent bibliometric analysis conducted by Hsu MH et al. underscores the substantial growth in publications concerning haptic technologies in dental education. This analysis reveals a noteworthy escalation from 13 publications between 2001 and 2010 to 72 publications between 2011 and 2022, giving promising pedagogical potential of these technologies.^7^ These haptic simulators offer a multitude of advantages, including objective measurements, self-assessment capabilities, instant feedback mechanisms, and the opportunity for students to repetitively practice exercises as needed. Consequently, they play a pivotal role in facilitating rapid and effective improvement of students' skills.^7^ The findings of this analysis show the significant attention haptic technology garners in dentistry, with promising pedagogical implications.^8^, ^9^, ^10^, ^11^, ^12^, ^13^, ^14^, ^15^, ^16^, ^17^, ^18^, ^19^, ^20^, ^21^, ^22^, ^23^ Haptic simulators offer several perspectives regarding their abilities to monitor student progress.^13^^,^^24^ Some researchers stated that students can markedly enhance their motor skills in a more efficient way through the haptic simulation, thanks to the immediate feedback it provides.^8^^,^^25^ Studies, such as the one conducted by Felszeghy S et al., demonstrated that integrating haptic and conventional simulations in fixed prosthodontics can improve the quality of the prosthetic preparation concerning the occlusal and axial reduction, the undercut management, and the preservation of adjacent teeth.^26^ Similarly, Coro-Montanet G et al. reported in their research that the most effective strategy for knowledge acquisition and skill learning involves the use of the Simodont haptic simulator, surpassing the efficacy of watching video tutorials or reading manual aids.^27^

Given their acknowledged educational values,^28^, ^29^, ^30^, ^31^, ^32^, ^33^, ^34^, ^35^, ^36^ it becomes pertinent to explore whether haptic simulators could aid in predicting the performances of novice dental students in conventional simulation scenarios. The limited studies addressing this question suggest a correlation between students' results in manual dexterity haptic exercises and their outcomes in preclinical assessments.^25^^,^^37^, ^38^, ^39^ One of such studies indicates that the occurrence of failures (poor progression or exercise not completed) during a single session of a complex haptic exercise—such as the incompletion within the allotted time or movements beyond specified boundaries—could forecast students' performances (preparation quality) in subsequent preclinical examinations.^37^ Hence, it seems plausible that students' initial performances on conventional simulators may align with their success (good progression) or failure (poor progression) rates in manual dexterity haptic exercises. Confirmation of this hypothesis could establish haptic simulators as predictive tools for assessing novice dental students' performances in conventional simulation scenarios.^40^ Consequently, a pedagogical approach focused on monitoring the frequency of failures in manual dexterity haptic exercises within specific timeframes could be introduced to early identify students facing manual dexterity challenges, enabling them to receive tailored support during the initial stages of their training. The objective of this prospective cohort pilot study was to ascertain whether correlations exist between: i) the number of failures in haptic manual dexterity exercises, ii) the haptic performance index, and iii) the quality of prosthetic preparation scores (reflecting student performances on plastic teeth). Our null hypothesis states that there is a correlation between the results of manual dexterity haptic tests, in terms of failure/success, and the quality scores of prosthetic preparation. Additionally, the study aims at evaluating students' perceptions regarding the use of the VirTeaSy Dental® haptic simulator.

## Materials and methods

The study was conducted on a cohort comprising 40 s-year dental students enrolled at the Dental Faculty of Nantes University in September 2022. The selection of the 40 participants was carried out using the simple random sampling method. This approach ensures a representative sample of the cohort, as each member of the reference population has an equal chance of being selected. To ensure impartiality, the list of students was anonymized, and a random number was assigned to each one. Subsequently, a person not affiliated with the research team randomly selected 40 numbers from the 77 assigned. These participants were considered as novice students. Indeed, they had no prior exposure to haptic or other conventional simulators. The average age of the participants was 19.7 ± 1.8 years. Ethical approval for the study protocol was obtained in July 12, 2022 from the Nantes University Ethics Committee (IRB number: IORG0011023), and each student provided oral consent before participating. Prior to the haptic exercises, students underwent 5 training sessions with the VirTeaSy Dental® simulator (VirTeaSy, HRV Simulation, Changé, France) in one week to acquaint themselves with its operations and functionalities. During each 20-min session, the student is introduced to the various virtual instruments, learns to differentiate between the different tissues (enamel, dentin, decayed tissue), and then practices simple manual dexterity exercises by milling the target area along a straight or curved line, or following a schematic axis. During these 20 min, the student could practice as many times as necessary and track their progress. Subsequently, one week later, they underwent testing, which involved completing a series of four manual dexterity haptic exercises in a single session, followed by an assessment on the conventional phantom.

### Manual dexterity exercises with VirTeaSy

**Figure 1:**
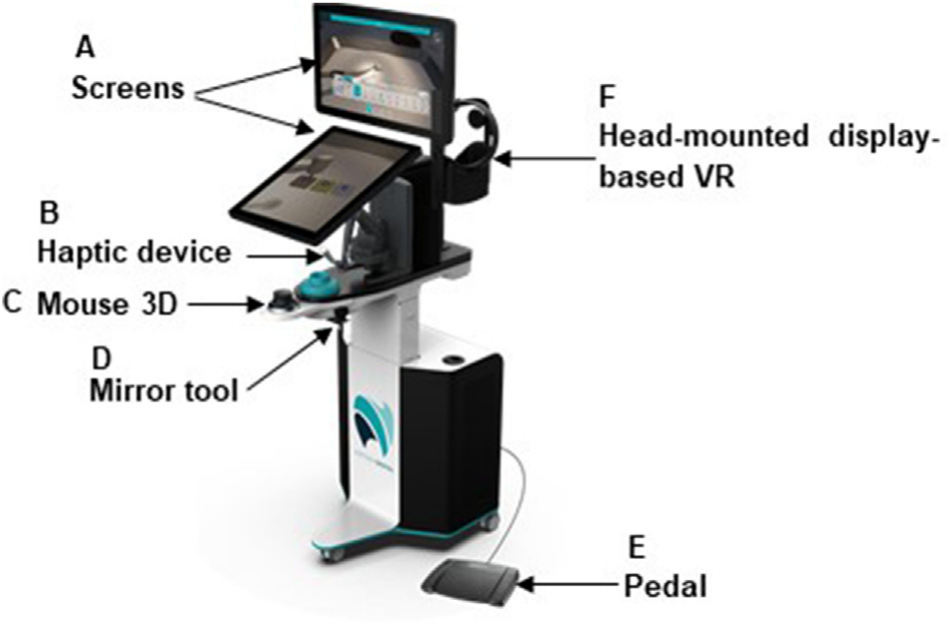
Haptic simulator VirTeaSy Dental® consists of various components: A) Screens connected to computer featuring touch interface for visualizing three-dimensional (3D) images. B) Haptic arm or physical handpiece enabling tactile interactions with simulator, providing real-time force feedback information. C) 3D mouse for controlling and interacting with simulator interface. D) Physical mirror linked to virtual mirror to facilitate visualization of dental procedures. E) Pedal for performing procedures, used to activate rotary instruments or other functions. F) Optionally, a virtual reality (VR) headset for full immersion in virtual environment.

The VirTeaSy simulator, developed by HRV Simulation, served as the primary tool in this study. This virtual learning environment for dental procedures offers force feedback and instantaneous responses, enhancing the learning experience. Prior studies already introduced this simulator.^19^^,^^21^^,^^41^ In brief, the VirTeaSy is made of a computer system linked to two screens, featuring a touch–screen interface for accessing the three-dimensional (3D) images, a haptic arm, a virtual reality (VR) headset (Oculus Quest, Meta Platforms Technologies, Menlo Park, CA, USA), a virtual mirror, a 3D mouse, and a foot pedal for activating rotating instruments (Fig. 1). The haptic arm, connected to a virtual contra-angle or turbine, delivers force feedback using the Geomagic Touch X Haptic Device (Geomagic Inc., Morrisville, NC, USA) and facilitates various dental procedures, such as tooth preparation, implant site drilling, and targeted area removal. Notably, the simulator can be used with or without a VR headset. For this study, the non-immersive haptic simulation, using the simulator without a VR headset was set.

**Figure 2:**
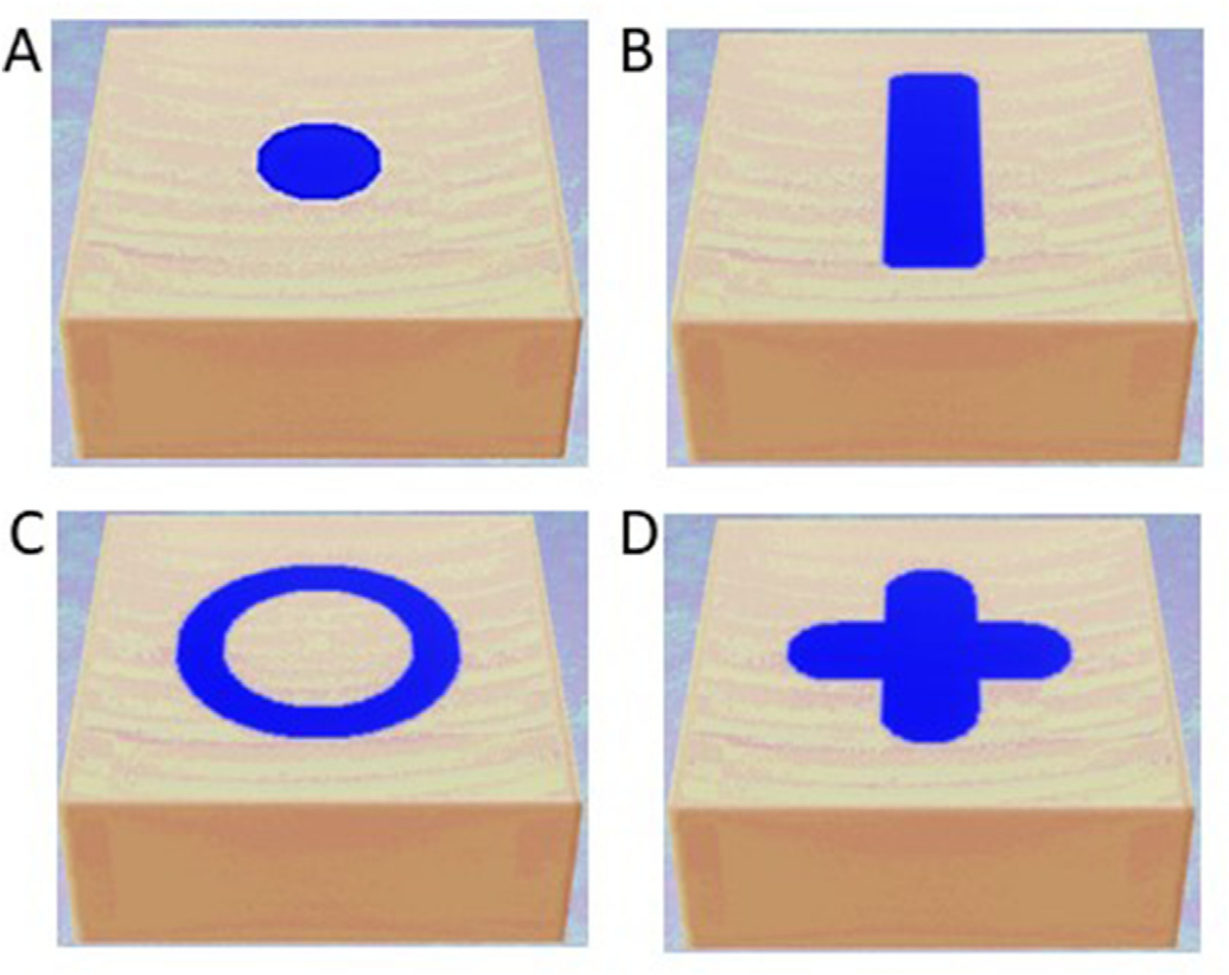
Haptic manual dexterity exercises. **(A)** Circle (Exercise 1). **(B)** Channel (Exercise 2). **(C)** Double circle (Exercise 3). **(D)** Cross (Exercise 4). The goal of these four exercises is to mailing a circle, a channel, a double circle, and a cross, by removing all the internal material (shown in violet), which is softer, without damaging the constrained walls or the base of the cavity. For each exercise, participants must accurately follow the contours of the prescribed drawing and create a cavity with a flat bottom and a constant depth of 2 mm. The shape's contour must precisely reflect the external form of the drawing and be constrained by the interface between hard and soft materials. The shape should be smooth and flat.

It should be remembered that there is a set of tests and tools to assess the manual dexterity of students, that is to say their precision on a given task.^39^^,^^42^, ^43^, ^44^, ^45^ Unfortunately, none of these tests (including haptic manual dexterity exercises) or tools have achieved consensus.^39^^,^^42^, ^43^, ^44^, ^45^ All the systems that exist are based on a single principle, that is to say the precision of the gesture, which reflects the level of dexterity, and by correlation its performance. So, the haptic manual dexterity exercises chosen to assess all participants are those already described in the literature,^41^ and included of four shapes with varying geometries. These shapes included a circle (Fig. 2A), a channel (Fig. 2B), a double circle (Fig. 2C), and a cross (Fig. 2D). Each exercise presents (i) a target area to be removed and (ii) margin areas (comprising lateral and lower edges) closely surrounding the target area, which participants are instructed to avoid as much as possible. During the session, 20 min in total was allocated to each participant, with prior instructions specifying that each exercise should be completed within 5 min. The objective for each exercise was to remove 90 % of the target zone while adhering to the predetermined width and depth limits. The 5-min time limit for each exercise was recommended by the Ziane-Casenave et al.^41^ If a participant failed to remove less than 90 % of the target zone within this timeframe, the test was deemed unsuccessful. Consequently, each student could have a number of failures between zero and four. Previous research demonstrated that repeating tasks, typically over an average of two exercises, unveils discernible discrepancies in manual dexterity proficiency between novice and expert participants. This evaluation considers factors such as the percentage of progress achieved and the time taken to complete each task.^38^^,^^41^^,^^46^, ^47^, ^48^

### Data collected from VirTeaSy

The following parameters were recorded on the simulator: (i) Total Time: This includes the duration of continuous rotation of the bur, whether the student is actively working or not; (ii) Drilling Time: Time spent specifically on tissue removal during the exercise; (iii) Overall Progression: Represents the overall percentage of tissue removed during the exercise; (iv) Precision: Reflects the student's ability to maintain the focus on the target area and remove tissue only at that level; (v) Internal Volume (IV): Indicates the volume of tissue removed by the participant within the target area; (vi) Outside Volume (OV): Represents the iatrogenic volume removed outside the target area by the participant. Data for these volumes were converted into percentages (%IV, %OV); (vii) Haptic Performance Index: Calculated using the formula: 100 - IV + OV (%).^41^

### Conventional simulator evaluation

Before the assessment on the conventional simulator, students underwent four training sessions aimed at instructing them on how to prepare plastic teeth for the insertion of a cast crown. Each session was held every Tuesday and lasted 2 h, resulting in the use of the conventional simulator over a 4-week period. At the beginning of each session, a senior staff member delivered a 10-min presentation to explain the objectives, preparation steps, and tissue reduction thicknesses. The goal was to ensure that the prepared tooth exhibited an appropriate contour shape and provided ample space for the cast crown insertion. The instructions used in the study by Hattori et al. were followed for each tooth preparation.^49^ The evaluation on the conventional simulator focused on preparing the right maxillary first premolar for a cast crown. Models were anonymized, and the preparation quality was assessed by a single evaluator. As a result, the evaluator could not identify the student to whom the model belonged, enabling blind evaluation. Scoring criteria established in previous literature were adopted for this study.^49^^,^^50^ These criteria encompassed: i) occlusal surface shape ii), cervical margin iii), preparation taper iv), occlusal reduction thickness v), overall reduction volume and vi), polishing. Each criterion was rated on a scale of 1 to 5 points (pts), resulting in a potential total score ranging from 6 to 30 for each student.

### Perceptions of VirTeaSy

The quantitative exploratory survey, conducted through a self-administered questionnaire (Table S1), involved all 40 students. The primary objective of the questionnaire was to collect feedback regarding the students' experience with the VirTeaSy simulator. The questionnaire consisted of three items (IT): IT1) Ease of use of VirTeaSy; IT2) Students' experiences when transitioning between the two simulators; and IT3) General impressions of using VirTeaSy.

### Statistical analysis

Statistical analyses were conducted using GraphPad® Prism software (GraphPad Prism Software Inc., San Diego, CA, USA) by an independent individual not affiliated with the research team. Qualitative variables were described using percentages and frequencies of each category, while quantitative variables were characterized by means, standard deviations, medians, and minimum and maximum values. Consistent with the approach in the Ziane-Casenave et al. study, a haptic performance index was employed.^41^ To examine the correlation between the number of failures in haptic exercises, student performance index in haptic exercises, and the score of prosthetic preparation quality on the conventional simulator, a Spearman rank correlation test was conducted following an assessment of the test's applicability. Additionally, Mann–Whitney U test was employed to compare the mean score of prosthetic preparation quality between students who failed the haptic tests and those who passed, while ANOVA with post hoc Tukey test was employed to compare all haptic parameters.

## Results

In this study, participation included 25 female students (62.5 %) and 15 male students (37.5 %). Demographic data, results from various haptic exercises, and the quality score for preparation on the conventional simulator are detailed in Table 1. Among the participants, 15 students (37.5 %) experienced at least one failure in the haptic exercises, while 25 students (62.5 %) had none. The mean performance index on the haptic simulator was 13.2 ± 6.4. However, an ANOVA followed by a post hoc Tukey test revealed no significant differences in overall progression rates or total time between the exercises (*P* > 0.05, Table 1). In contrast, there was a notable variation in the mean rates of internal volume removed across the exercises, with statistically significant differences for all comparisons (*P* < 0.05, Table 1). Conversely, data on external volume removed, drilling time, and average precision scores showed high variability between exercises. Significant differences in external volume removed were observed between exercises 1 and 2, 1 and 3, 1 and 4, 2 and 3, and 3 and 4 (*P* < 0.05, Table 1). The mean accuracy score also showed significant differences between exercises 1 and 3, 2 and 3, and 3 and 4 (*P* < 0.05, Table 1).

**Table 1:** Demographic data, haptic exercise results and prosthetic preparation quality score on conventional simulator.

Demographic data (N = 40)						
Age mean ± SD (Min-Max)	19.7 ± 1.8 (18–27)					

Sex % women (n/N); median (Q1; Q3)		62.5 % (25/40); 19 (19; 20)				

**Haptic simulator parameters**		**Exercise 1 N** = **40**	**Exercise 2 N** = **40**	**Exercise 3 N** = **40**	**Exercise 4 N** = **40**	**All exercises**

Total time (secondes)	Min-Max	[125; 335]	[124; 331]	[176; 359]	[123; 340]	[177.7; 327]
	Mean ± SD	268.8 ± 55.4^a^	250.6 ± 51.9^a^	274.4 ± 49.6^a^	263.5 ± 52.6^a^	264.3 ± 40.4
	Median [Q1; Q3]	287.5 [242; 312]	247.5 [215.2; 298]	301.5 [231.5; 314]	269.5 [244.2; 302.7]	273.7 [236.9; 299.5]
Drilling time (secondes)	Min-Max	[28; 231]	[43; 265]	[39; 231]	[14; 277]	[58.5; 251]
	Mean ± SD	99.3 ± 46.7^a^	123.9 ± 53.7^a^	116.4 ± 51.2^a^	141.4 ± 53.6^b^	99.3 ± 45.4
	Median [Q1; Q3]	95 [59.3; 125]	116 [83.5; 160.7]	108.5 [78; 160]	132.5 [108; 180]	109.9 [87.9; 153.4]
Target progression (%)	Min-Max	[74.5; 96.5]	[85.3; 98.6]	[76.2; 96.7]	[84.1; 94.3]	[84.0; 94.4]
	Mean ± SD	90.1 ± 4.1^a^	90.8 ± 1.9^a^	89.2 ± 3.6^a^	90.0 ± 1.5^a^	90.0 ± 1.8
	Median [Q1; Q3]	90.6 [90.0; 91.6]	90.4 [90.2; 91]	90.6 [90.0; 91.6]	90.0 [90.1; 90.5]	90.3 [89.5; 90.8]
Accuracy (%)	Min-Max	[44.6; 99.9]	[48.7; 99.6]	[53.1; 93.5]	[58; 97.7]	[67.8; 94.6]
	Mean ± SD	84.7 ± 13.4^a^	80.6 ± 12.5^ab^	73.8 ± 10.2^bc^	87.0 ± 7.8^ac^	81.5 ± 7.2
	Median [Q1; Q3]	89.6 [80.1; 95.2]	89.6 [80.1; 95.2]	74.5 [67.3; 82.0]	87 [83.4; 94.2]	82.0 [76.1; 86.6]
Inside volume (%IV)	Min-Max	[17.6; 22.8]	[48; 55.5]	[50.4; 64]	[65.6; 73.5]	[46.3; 52.8]
	Mean ± SD	21.3 ± 0.9^a^	51.1 ± 1.1^b^	59.1 ± 2.4^c^	70.2 ± 1.2^d^	50.4 ± 1.0
	Median [Q1; Q3]	21.4 [21.3; 21.6]	50.9 [50.8; 51.2]	59.6 [59.3; 59.8]	70.3 [70.2; 70.5]	50.5 [50.0; 50.8]
Outside volume (%OV)	Min-Max	[0; 26.4]	[0.2; 54]	[3.8; 52.2]	[1.6; 51.1]	[3.2; 29.6]
	Mean ± SD	4.7 ± 5.7^a^	14.2 ± 12.9^ab^	22.6 ± 12.0^b^	11.2 ± 8.8^ab^	13.2 ± 6.4
	Median [Q1; Q3]	2.5 [1.1; 5.3]	10.7 [6.7; 18.7]	20.4 [12.6; 28.1]	10.5 [4.4; 14.1]	11.8 [8.7; 15.7]
Haptic skill index (%)	Min-Max	[0.1; 55.4]	[0.4; 51.3]	[6.5; 46.9]	[2.3; 42]	[3.2; 29.6]
	Mean ± SD	15.3 ± 13.4^a^	19.4 ± 12.5^ab^	26.2 ± 10.2^bc^	13.0 ± 7.8^ac^	13.2 ± 6.4
	Median [Q1; Q3]	10.4 [4.8; 19.9]	17.4 [11.7; 26.9]	25.4 [17.9; 32.6]	13.0 [5.8; 16.6]	18.0 [13.4; 23.9]
Successful completion of haptic simulator exercises if the overall progress reaches 90 % in 5 min.	Success	32 (80.0 %)	38 (95.0 %)	31 (77.5 %)	33 (82.5 %)	25 (62.5 %)
	Failed	8 (20.0 %)	2 (5.0 %)	9 (22.5 %)	7 (17.5 %)	15 (37.5 %)
**Preparation quality score for conventional simulator (N = 40)**				**Min-Max**	**Mean ± SD**	**Median [Q1;Q3]**

Occlusal morphology				[1; 4]	2.2 ± 0.9	2 [1; 3]
Cervical margin				[1; 4]	1.8 ± 0.8	2 [1; 2]
Overall taper				[1; 5]	2.4 ± 1.0	2 [1.2; 3]
Occlusal reduction				[1; 5]	1.9 ± 0.9	2 [1.5; 2.3]
Overall morphology				[1; 4]	1.7 ± 0.5	2 [1; 2]
Polishing				[1; 4]	2.1 ± 1.0	2 [1; 3]
**Min-Max, mean score and median**				[6; 23]	12.3 ± 4.5	12.5 [9; 14]
Average score between successful and failed haptic tests				Success (n = 25)	13.72 ± 4.76^a^	(*P* < 0.05)
				Failed (n = 15)	10.66 ± 3.69^b^	
**Mean time (in minutes) of prosthetic preparation on conventional simulator**				[45; 92]	73.5 ± 10.5	74.5 [65.7; 80]

Max, maximum; Min, minimum; N, number of participants; SD, standard deviation. Identical letters within each column (horizontal) indicate no statistically significant differences (*P* > 0.05), whereas differing letters signify statistically significant differences between haptic exercises parameters or mean prosthetic quality score (*P* < 0.05). Exercise 1 (Manual dexterity circle): This exercise involves drilling along the contour of a simple circle, focusing on precision, fluidity, and movement consistency to improve manual control. Exercise 2 (Manual dexterity channel): This exercise requires drilling within a narrow channel, ensuring not to touch the edges, aimed at improving dexterity. Exercise 3 (Manual dexterity double circle): This exercise involves following two concentric circles, requiring increased coordination to maintain a precise trajectory between the two shapes. Exercise 4 (Manual dexterity cross): This exercise consists of drilling along the lines of a cross, demanding precise movement control in multiple directions.

**Figure 3:**
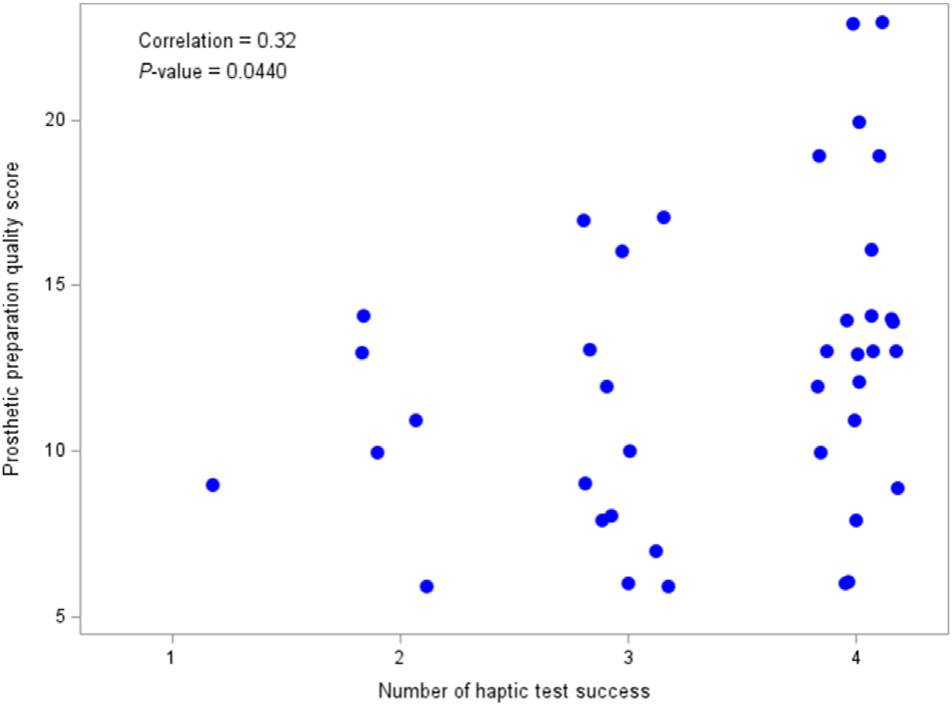
Correlation between number of successes in haptic exercises and score of prosthetic preparation quality on conventional simulator.

**Figure 4:**
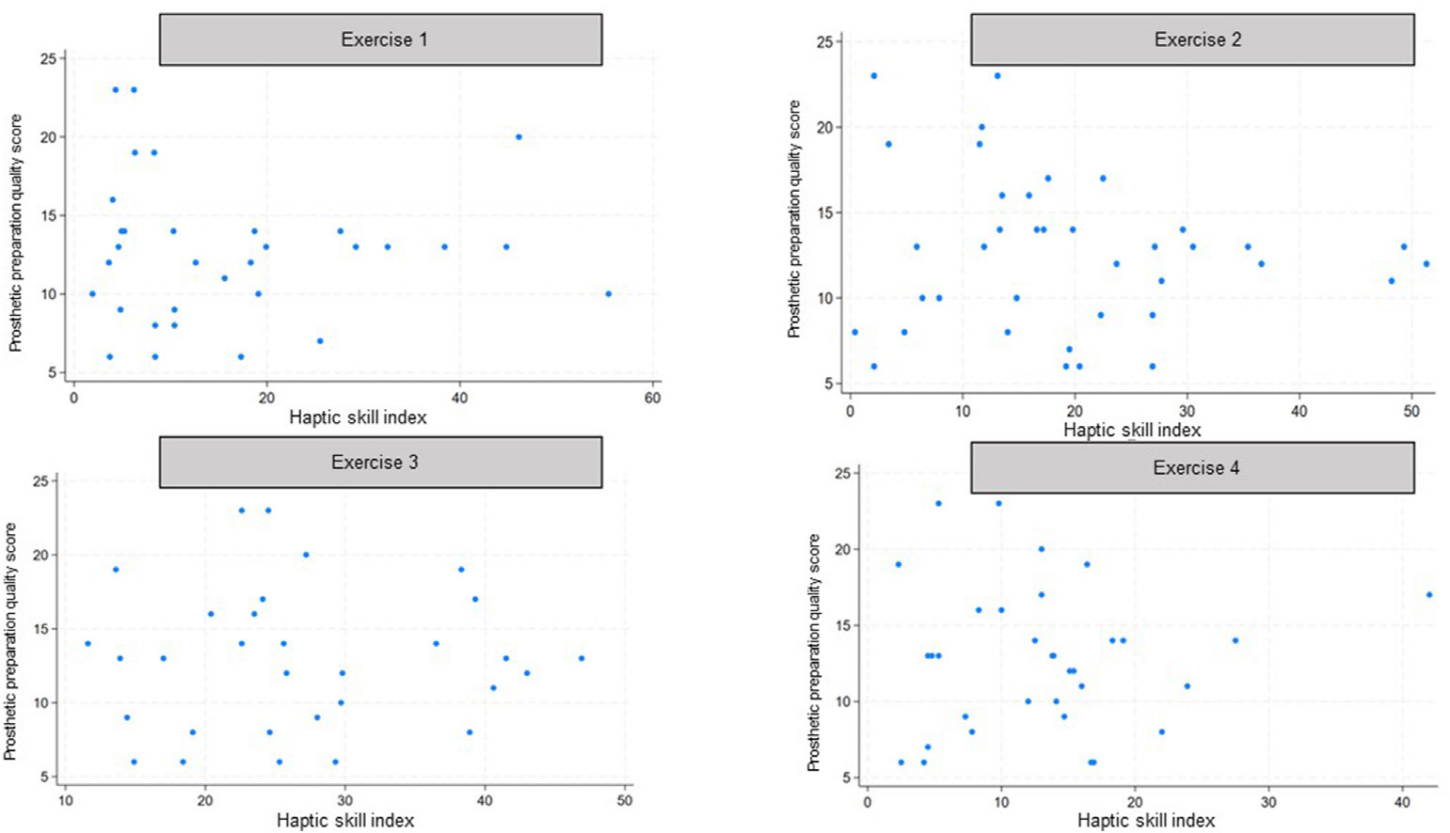
Correlation between student's performance index in haptic exercises and score of prosthetic preparation quality on conventional simulator.

There is a significant correlation between the number of successful haptic exercises and the quality score of prosthetic preparation on the conventional simulator (*P* < 0.05) (Fig. 3). Specifically, students who succeeded in all haptic exercises (n = 25) achieved higher scores on the conventional simulator (13.72 ± 4.76), whereas those who experienced at least one failure (n = 15) scored lower (10.66 ± 3.69). This difference in mean scores (3.06) is statistically significant (*P* < 0.05; OR, 1.19; CI, 1.02–1.44) (Table 1). However, the scatter plot analysis reveals no linear relationship between the students' performance index in haptic exercises and the quality score of prosthetic preparation on the conventional simulator (Fig. 4, Table 2).

**Table 2:** Spearman correlation coefficient between student performance index on haptic exercises and the full-contour preparation quality score on conventional simulator.

	Haptic skill index				
	Exercise 1	Exercise 2	Exercise 3	Exercise 4	Exercises 1, 2, 3, 4
Preparation quality score on conventional simulator					
Correlation coefficient	0.02	−0.11	0.05	0.11	0.05
*P*-value	0.89	0.48	0.73	0.49	0.73
N	40	40	40	40	40

N, number of participants.

The Haptic skill index is a quantitative measure used to evaluate fine motor skills during simulated tasks with haptic feedback (force or touch feedback). It assesses parameters such as internal and external volume for each of the haptic exercises 1, 2, 3, and 4. There is no significant correlation between the students' performance indices on the haptic exercises and the quality of their preparation on the conventional simulator. The correlation coefficients are all very low (close to 0), and the high p-values (>0.05) suggest the absence of a statistically significant relationship.

Thirty-eight out of the 40 students completed the questionnaire, while two participants did not finish it. For IT1, 50 % of students found the VirTeaSy easy or very easy to use, while 39.5 % found it neither easy nor difficult. For IT2, 50 % of students reported a needing time to adapt to the conventional simulator, whereas 42.1 % had no particular problems regarding the transition. For IT3, 55.3 % of students found the VirTeaSy useful for learning the milling gesture, 81.6 % enjoyed using it, and 68.4 % expressed a desire to use it again. Despite these positive aspects, students' opinions were divided, and the difference in ergonomics such as working posture, positioning around the simulator, support point, *e.g*. between the haptic and conventional simulators discouraged seamless switching between the two. Details of these results are presented in Supplementary Table S2.

## Discussion

Haptic simulators can play a crucial role in predicting students' performances in conventional simulation.^51^ The null hypothesis—that a correlation exists between the results of haptic manual dexterity exercises, in terms of failure, and the prosthetic preparation quality score—was accepted. Our study clearly demonstrates a correlation between the number of failures in haptic exercises and the quality of prosthetic preparation on the conventional simulator. This emphasizes the importance of these exercises in identifying students with manual dexterity difficulties and tailoring their training accordingly. Students who failed at least one haptic exercise scored lower on the conventional simulator compared to those who never failed. These findings suggest that students who struggle with haptic exercises likely have manual dexterity issues, which adversely affect their prosthetic preparation quality on the conventional simulator. Our results align with those of Urbankova et al., who found that the number of failures during a single session of complex haptic exercises correlates with student performances in a preclinical setting.^37^^,^^52^ The same authors reported similar results in their recent publication, noting that 9 out of 30 novice students who failed the haptic tests at least once, performed worse on the preclinical restorative dentistry examination.^39^ According to these authors, haptic test success predicts preclinical performances with a sensitivity of 72 % and a specificity of 92 %. Consequently, the failure in haptic tests can play a decisive role in the early identification of students with manual dexterity issues, allowing for targeted support during the initial stages of preclinical training. Haptic simulators are valuable tools for predicting students' preclinical performances, facilitating better differentiation and support for those requiring additional pedagogical assistance. In a similar vein, Al-Saud LM et al. concluded that students who scored high on the haptic simulator were approximately 10 times more likely to perform well on the conventional simulator.^25^ Although our study differs from those of Urbankova et al. and Al-Saud LM et al. in terms of the type of haptic simulator used, the nature of the preclinical examination, and the haptic exercises performed, the data consistently show that the number of failures in haptic exercises is correlated with lower student performance scores on the conventional simulator. Consequently, failure in haptic tests is a valuable criterion for the early identification of students with manual skill difficulties, with a particular emphasis on haptic exercises in indirect vision, as suggested by David J et al.^53^ To date, only the works of Urbankova A et al. and Al-Saud LM et al. has focused on predicting the performances of novice students.^25^^,^^37^, ^38^, ^39^ This makes challenging the comparison of our results on this parameter with those of other studies. Most existing research focused on the ability of haptic simulators to differentiate between student and expert profiles based on their preclinical or clinical experiences.^41^^,^^51^^,^^53^ The findings from these studies, summarized in our recent systematic review, indicate that the score of a haptic manual dexterity test can, to some extent, distinguish users according to their initial performances level.^51^ However, some authors, such as Ziane-Casenave et al., concluded that the scores of haptic exercises in direct vision of the VirTeaSy simulator did not make possible the differentiation between users of different levels of expertise.^41^ According to Joseph D et al., this type of direct vision exercise, considered as too easy, was not discriminative. The authors recommend complex haptic exercises in indirect vision, which could better distinguish between different student profiles with varying preclinical and clinical levels.^53^ In their systematic review, Patil S et al. concluded that data collected by the Simodont haptic simulator could distinguish students based on their level of manual skill and predict their clinical performances.^8^ By comparing the results of experienced and novice students, Mirghani et al. indicated that Simodont was sensitive to performance differences between the two profiles and could therefore be used to measure student performances.^48^ Other authors, such as Osnes C et al., reached similar conclusions, noting that clinicians outperformed novices in terms of accuracy and overall performances during a haptic exercise on Simodont.^54^ Additionally, it is interesting to note that the haptic performance index showed no significant correlation with the prosthetic preparation quality. This suggests that the haptic performance index may not be a discriminating parameter in this specific context. In the study by Ziane-Casenave et al., the haptic performance index did not distinguish participants according to their level of manual dexterity.^41^ Consequently, the haptic simulator performance index appears to be a parameter of limited value and should not be prioritized for evaluating students' manual skills.

It's promising to note that the majority of students find the VirTeaSy haptic simulator useful for learning the drilling gesture. These perceptions reflect a certain enthusiasm among students and indicate that the simulator contributes positively to their learning of the milling gesture. These results align with previous studies, which consistently shown that novice dental students perceive haptic simulators as valuable tools for enhancing their manual dexterity.^10^^,^^26^^,^^51^^,^^55^, ^56^, ^57^, ^58^ It's worth considering the findings of Daud A et al., which suggested that haptic simulation should complement rather than replace conventional training on phantoms.^58^ The authors recommend optimizing the use of haptic technology by integrating it into dental education, provided that trainers accompany its use to maximize its effectiveness during preclinical training.^58^ To fully harness its pedagogical potential, it is advised that students engage in enough practice to ensure the transferability of skills to real situations.^8^ Some authors, like Urbankova et al., suggest allocating 8 h of haptic simulation in the early stages of preclinical dental education to enhance student performance.^59^ Our study highlights certain difficulties encountered by students, such as gripping the haptic arm and maintaining the required working posture imposed by the simulator. Additionally, challenges may arise from computer bugs occurring during exercises, making it difficult to control the drill. In summary, factors such as the grip of the haptic arm and the working position seem to influence the ease of use of the VirTeaSy simulator. Moreover, an adjustment period is often necessary when transitioning from the haptic arm to the turbine on the conventional simulator. Continuous improvements in tissue density and cutter control are necessary in the VirTeaSy simulator to enhance simulation realism and mitigate the various difficulties encountered by students. Furthermore, although the models were anonymized, it should be noted that the evaluation of the preparations was carried out by the same person who conducted the experiment, and is therefore based solely on their subjective opinion, making the method open to criticism. The widely recommended inter-judge evaluation could not be implemented. Furthermore, due to the low correlation coefficients observed in all tests, it is challenging to meaningfully discuss the quantitative results.

The findings of this study underscore the predictive value of the number of failures in haptic manual dexterity exercises for student performances on the conventional simulator. This highlights its potential role in the early identification of novice dental students facing manual dexterity challenges and tailoring their preclinical training accordingly. While the VirTeaSy haptic simulator garners enthusiasm for most students in learning the milling gesture, a transition period is essential when switching from VirTeaSy to the conventional simulator. Technical advancements are imperative to develop more realistic and sophisticated haptic simulators.

## Declaration of competing interest

The authors have no conflicts of interest relevant to this article.
