# Supplementary material for: Predicting novice dental students' performances in conventional simulation: A prospective pilot study using haptic exercises
Source: J Dent Sci. 2024 Nov 12;20(2):943–52. doi: 10.1016/j.jds.2024.10.023 (PMC11993030; doi:10.1016/j.jds.2024.10.023)
Supplement: Multimedia component 1 [file mmc1.docx]

Table S1. Students’ perception regarding the use of VirTeaSy Dental^®^ VR-haptic simulator during a manual dexterity exercise.

| Item 1 (IT1): Virteasy Dental® easy to use  No. of respondents | | | | | | | |
| --- | --- | --- | --- | --- | --- | --- | --- |
|  | | Very easy | Easy | | Moderately | Difficult | Very difficult |
| Q1) How do you feel about the Virteasy simulator on the following points? [Using the Virteasy simulator] % (n/N) | |  |  | |  |  |  |
| Q2) How would you rate the Virteasy simulator on the following points: [Grip (manipulation) of the instrument (haptic arm)]? % (n/N) | |  |  | |  |  |  |
| Q3) How do you feel about the working position imposed by the simulator? % (n/N) | | | | | | | |
| I adapted easily | | | | | |  | |
| It took me a while to adopt a suitable position | | | | | |  | |
| I have no opinion | | | | | |  | |
|  | | | | | | | |
| Item 2 (IT2): How students felt when they switched from the Virteasy Dental® simulator to a conventional simulator  No. of respondents | | | | | | | |
| Q1) What was it like to switch to a conventional simulator after using the Virteasy simulator? % (n/N) | | | | | | | |
| I needed time to adapt | | | | | |  | |
| I had difficulties | | | | | |  | |
| I had no particular problems | | | | | |  | |
| Q2) How would you rate your experience with the conventional simulator?  [When I switched to the conventional instrument (turbine), I needed time to adapt]. % (n/N) | | strongly agree | somewhat agree | | moderately agree | somewhat disagree | strongly disagree |
|  |  |  |  | |  |  |  |
| Q3) When I switched to the conventional instrument (turbine), I felt at ease. | |  |  | |  |  |  |
|  | | | | | | | |
| Item 3 (IT3): General impressions of Virteasy Dental^®^ use  No. of respondents | | | | | | | |
|  | strongly agree | | | somewhat agree | moderately agree | somewhat disagree | strongly disagree |
| Q1) What are your general impressions of the following points? [The Virteasy simulator was useful in helping me learn the milling gesture] | |  |  | | | | |
| Q2) I liked using the Virteasy simulator | |  |  | | | | |
| Q3) I'd like to use the Virteasy simulator again during training | |  |  | |  |  |  |

IT, item; Q, question; No. number.
