# Supplementary material for: Predicting novice dental students' performances in conventional simulation: A prospective pilot study using haptic exercises
Source: J Dent Sci. 2024 Nov 12;20(2):943–52. doi: 10.1016/j.jds.2024.10.023 (PMC11993030; doi:10.1016/j.jds.2024.10.023)
Supplement: Multimedia component 2 [file mmc2.docx]

Table S2. Students’ perception regarding the use of VirTeaSy Dental^®^ VR-haptic simulator during a manual dexterity exercise.

| Item 1 (IT1): Virteasy Dental^®^ easy to use  No. of respondents (N =38) | | | | | |
| --- | --- | --- | --- | --- | --- |
|  | Very easy | Easy | Moderately | Difficult | Very difficult |
| Q1) How do you feel about the Virteasy simulator on the following points? [Using the Virteasy simulator] % (n/N) | 2.6 % (1/38) | 47.4 % (18/38) | 39.5 % (15/38) | 7.9 % (3/38) | 2.6 % (1/38) |
| Q2) How would you rate the Virteasy simulator on the following points: [Grip (manipulation) of the instrument (haptic arm)]? % (n/N) | 5.3 % (2/38) | 26.3 % (10/38) | 52.6 % (20/38) | 13.2 % (5/38) | 2.6 % (1/38) |
| Q3) How do you feel about the working position imposed by the simulator? % (n/N) | | | | | |
| I adapted easily | | | | 39.5 % (15/38) | |
| It took me a while to adopt a suitable position | | | | 57.9 % (22/38) | |
| I have no opinion | | | | 2.6 % (1) | |
|  | | | | | |
| Item 2 (IT2): How students felt when they switched from the Virteasy Dental® simulator to a conventional simulator  No. of respondents (N =38) | | | | | |
| Q1) What was it like to switch to a conventional simulator after using the Virteasy simulator? % (n/N) | | | | | |
| I needed time to adapt | | | | 50.% (19/38) | |
| I had difficulties | | | | 7.9 % 3/38 | |
| I had no particular problems | | | | 42.1 % (16/38) | |
| Q2) How would you rate your experience with the conventional simulator?  [When I switched to the conventional instrument (turbine), I needed time to adapt]. % (n/N) | strongly agree | somewhat agree | moderately agree | somewhat disagree | strongly disagree |
|  | 23.7 % (9/38) | 36.8 % (14/38) | 21 % (8/38) | 13.2 % (5/38) | 5.3 % (2/38) |
| Q3) When I switched to the conventional instrument (turbine), I felt at ease. | 7.9 % (3/38) | 50 % (19/38) | 34.2 % (13/38) | 5.3 % (2/38) | 2.6 % (1/38) |
|  | | | | | |
| Item 3 (IT3): General impressions of Virteasy Dental^®^ use  No. of respondents (N =38) | | | | | |
| Q1) What are your general impressions of the following points? [The Virteasy simulator was useful in helping me learn the milling gesture] | 7.9 % (3/38) | 47.4 % (18/38) | 31.6 % (12/38) | 10.5 % (4/38) | 2.6 % (1/38) |
| Q2) I liked using the Virteasy simulator | 47.4 % (18/38) | 34.2 % (13/38) | 10.5 % (4/38) | 5.3 % (2/38) | 2.6 % (1/38) |
| Q3) I'd like to use the Virteasy simulator again during training | 36.8 % (14/38) | 31.6 % (12/38) | 13.2 % (5/38) | 15.8 % (6/38) | 2.6 % (1/38) |

Q: Question; No.: Number; IT: Item. For IT1, 50% of students found the simulator easy or very easy to use, while 39.5% found it neutral in terms of difficulty, and only 10.5% considered it difficult or very difficult. Regarding the handling of the haptic arm, 52.6% of students found it neither easy nor difficult, 15.8% found it very difficult, and 31.6% found it easy or very easy. Concerning the working position imposed by the simulator, 57.9% of students reported that it took them a long time to adopt a suitable position, whereas 39.5% adapted easily. These findings suggest that opinions on the ease of use of the haptic simulator are varied, with the grip of the haptic arm and the working position being key factors influencing user experience.

For IT2, half of the students reported needing time to adjust from the haptic simulator to the conventional simulator, while only 7.9% encountered difficulties and 42.1% experienced no issues. Specifically, 60.5% of students needed time to adapt when transitioning from the haptic arm to the turbine, compared to 18.5% who did not. Despite the majority requiring an adjustment period, 57.9% felt comfortable during the transition, while 34.2% were neutral and 7.9% felt uncomfortable. These results highlight a divergence in students' experiences with the transition from haptic to conventional simulation, with most requiring adaptation but over half feeling comfortable during the shift.

For IT3, 55.3% of students found the VirTeaSy Dental® simulator useful for learning the milling gesture, 31.6% considered it moderately useful, and 13.1% found it not beneficial. A significant majority, 81.6%, enjoyed using the simulator, and 68.4% expressed a desire to use it again, compared to 18.4% who did not. These findings indicate that the simulator is highly valued and beneficial for most students in acquiring milling skills, though opinions are divided, and the ergonomic differences between haptic and conventional simulations may hinder smooth transitions.
